# Positive youth development attributes and cyberbullying victimization among Chinese middle school students: A longitudinal moderated mediation model involving internet gaming disorder and depression

**DOI:** 10.1371/journal.pone.0287729

**Published:** 2023-06-30

**Authors:** Xiong Gan, Pinyi Wang, Guoxing Xiang, Xin Jin

**Affiliations:** 1 Department of Psychology, College of Education and Sports Sciences, Yangtze University, Jingzhou, China; 2 Department of Psychology, College of Education and Sports Sciences, Yangtze University College of Technology and Engineering, Jingzhou, China; University of Study of Bari Aldo Moro, ITALY

## Abstract

As an extension of traditional bullying, cyberbullying emerges with the increasing popularity of the internet, and seriously affects the health of students. However, fewer studies have explored the potential influencing mechanisms of cyberbullying victimization from a positive psychology perspective. Therefore, based on the positive youth development theory, this study will explore the potential mediator and moderator in the relationship between positive youth development (PYD) attributes and cyberbullying victimization through a longitudinal design. 719 students (*M*_age_ = 15.95 years, *SD* = 0.76, 45.2% boy) participated in the study and completed self-report questionnaires on relevant variables. The result found that students’ level of PYD significantly and negatively predicted the level of cyberbullying victimization. Meanwhile, SEM analysis showed that PYD influenced individuals’ cyberbullying victimization by affecting their internet gaming disorder (IGD), while depression levels moderated the relationship between PYD and IGD. This study examines cyberbullying victimization from a positive psychology orientation, with potential prevention and intervention value.

## 1. Introduction

According to the China Internet Network Information Center’s 50th Statistical Report, as of June 2022, the number of internet users in China had reached 1.051 billion, with 13.5% of internet users between the ages of 10 and 19 [1]. With the rising popularity of the internet, the issue of cyberbullying among adolescents is becoming an extremely incisive social problem [2]. Although the bullying problem in mainland China has improved in recent years [3], it is still a hot issue of widespread concern among relevant researchers due to its inherent complexity and its serious impact on the physical and mental health of youth. For example, it has been shown that psychological distress (e.g., anxiety) predicts adolescent victimization [4], but bullying experiences have also been shown to influence adolescent psychological distress, which in turn plays an important role in non-suicidal self-injury [5]. As an extension of traditional bullying behavior on the internet, cyberbullying is defined as an intentional, aggressive, and repetitive behavior perpetrated by a more powerful individual against someone more vulnerable through the use of technology such as the internet, social media, and cellular phones [6]. Because of the medium of the internet, cyberbullying is more spreadable than traditional bullying [7,8]. Relevant studies show that cyber deviance [9], especially cyberbullying [10], is very common in the social context of mainland China as well as Taiwan and Hong Kong. As a result, this behavior can cause immeasurable harm to the victims. Many studies have confirmed the negative impact of cyberbullying behavior on adolescents [6], especially on the victims [7,11]. For example, Chen used structural equation modeling analysis through a cross-societal probability sample survey to demonstrate a significant direct association between cyberbullying victimization and psychological distress [12]. In addition, cyberbullying victimization has been shown to significantly influence adolescents’ emotions [7,13] and academic performance [14]. Therefore, the increased attention to cyberbullying victimization contributes to the healthy physical and mental development of students. However, previous researchers have mostly explored the negative effects of cyberbullying victimization and have paid less attention to its influencing factors and underlying influencing mechanisms. In addition, researchers have focused more on cyberbullying victimization as a problem behavior and explored how to manage it, and less on how to create a positive environment to have a long-lasting impact on it. Therefore, in order to better understand adolescent cyberbullying victimization and provide prevention and intervention value, this study intends to investigate the effects of positive youth development attributes on adolescent cyberbullying victimization and the potential mediator (i.e. internet gaming disorder) and moderator (i.e. depression) involved.

Because cyberbullying behavior uses the internet as a medium and has only been gaining attention in recent years [15,16], research on this behavior is not yet as rich as that on offline bullying (e.g., school bullying). In addition, with the emerging trend of positive psychology, research on the factors influencing cyberbullying victimization can shift from previous risk factors (e.g., low socioeconomic status, introverted personality traits) [8] to protective factors (e.g., positive youth development attributes). According to D’Urso et al., being bullied is part of a broader, complex system of adolescent psychological and social development [17]. However, previous research on bullying does not usually consider such a wide range of relevant factors and rarely places bullying in a specific context. Positive youth development theory [18] indicates that by providing adolescents with some external developmental resources and enhancing adolescents’ own internal resources, adolescents can develop well and achieve effective prevention or reduction of problem behaviors. Taking a positive youth development perspective on youth cyberbullying victimization not only places the behavior in a broader context, but also emphasizes the potential for youth development rather than the "possible" deficits, marking a fundamental change in the understanding of the nature of youth development. Based on revealing the influencing factors of adolescent cyberbullying victimization, the exploration of its mediating and moderating variables will also help us better understand the influence pathways and boundary conditions of positive youth development attributes and provide new perspectives for prevention and intervention measures.

### 1.1. PYD attributes and cyberbullying victimization

Positive youth development (PYD) is an optimal development that strives for full, healthy, and successful development [14], which focuses on the strengths and potentials of youth. According to positive youth development theory [18], by providing youth with some external developmental resources (e.g., family support, community resources, etc.) and improving youth’s internal resources (e.g., self-efficacy, future beliefs, psychological resilience, etc.), youth can achieve positive development and achieve effective prevention or reduction of problem behaviors due to less exposure to risky environments [19,20]. For example, researchers have found significant direct effects of parental rejection on school victimization and school bullying through large samples [21]. School engagement has also been shown to be a significant negative predictor of victimization in school and cyberspace [22]. In addition, a good relationship with parents has been shown to be a protective factor against bullying [23]. Therefore, it is consistent with the comprehension theoretical model of problem behavior (i.e., family, peers, school, and community all have protective and risk factors that can have a direct impact on problem behavior) [24], for individuals with lower levels of PYD, problem behavior is relatively high due to the lack of contextual resources.

Previous researchers have illustrated the relationship between PYD attributes and school bullying. For example, Hui et al. [25] used the positive youth development paradigm to explore prevention programs for school bullying. In addition, D’Urso et al. [17] explored the dynamic relationship between PYD attributes and bullying through a longitudinal study design and found that PYD attributes had a significant predictive effect on subsequent bullying victimization. However, research on PYD attributes and cyberbullying victimization needs to be deepened. As one of the branches of bullying, cyberbullying and school bullying have very high similarity and commonality [26]. Thus, combining positive youth development theory and the comprehension theoretical model of problem behavior, PYD may be a significant predictor of cyberbullying victimization.

### 1.2. IGD as a mediator

Internet gaming disorder (IGD) is defined as an individual’s uncontrollable, excessive and compulsive play of online games that results in impaired physical, psychological and social functioning [27]. IGD is one of the serious adverse outcomes that occur in the younger generation [28]. According to recent studies, Chinese adolescents have such a high prevalence of IGD, ranging from 2.97% to 13% [29,30]. As consequence, IGD in adolescents has become a major concern in the relevant academic fields nowadays. On the one hand, Researchers showed that IGD causes a variety of negative effects in adolescents, including anxiety, insomnia, poor academic performance, and so on [31–34]. Adolescents with higher levels of IGD spend more time in online environments and are more likely to suffer from adverse environmental repercussions. A strong relationship between internet addiction and increased cyberbullying has been shown [35]. On the other hand, the positive youth development theory [18] focuses on the strengths and potentials of youth, nurturing youth as a resource. It has been shown that adolescents with different PYD assets have different levels and types of problem behaviors, such as IGD [36,37]. Shek demonstrates that PYD intervention is a proactive intervention strategy to address adolescent IGD [38]. According to self-determination theory [39], individuals with higher levels of PYD have higher levels of internal resources and refined internal motivation to better identify the advantages and disadvantages of networks [40]. Therefore, students with higher levels of PYD attributes can also clearly identify the negative effects of IGD as a way to regulate their internet use. For example, Yu and Shek, using Hong Kong students as their participants, found that specific PYD attributes were important protective factors for internet disorder [41]. Dou and Shek’s study not only confirmed the predictive effect of PYD attributes on internet addiction at the cross-sectional level, but also demonstrated significant effects of PYD at the longitudinal level [40].

According to developmental contextualism [42], human development is achieved through the continuous interaction between an individual and the context in which they are placed. Students with IGD spend more time in the internet than those without this disorder [43]. As a result, they may have more opportunities to be victimized by cyberbullying. Under this model, PYD not only directly influences cyberbullying victimization but may also influence cyberbullying victimization in conjunction with the internet context. And research has shown that PYD interventions influence youth bullying victimization by improving the context [16,25]. Therefore, we believe that PYD attributes may have an impact on cyberbullying victimization by influencing IGD.

### 1.3. Depression as a moderator

Although PYD attributes may affect IGD, it does not influence adolescents equally. It should be noted that adolescent development is the result of a combination of multiple factors, not only environmental factors, but also individual factors, and even the interaction of two factors. In other words, the influence of environmental factors on adolescent development is different for different individuals [42]. Depression is defined as a negative emotional experience resulting from an individual’s feeling of inability to cope with external stressors [44]. Depression as a negative emotion has become a major risk factor affecting the physical and mental health of adolescents due to its huge impact on the individual’s physiology and psychology [44]. Previous studies have shown that individuals’ negative emotions are influenced not only by their acquired environment but also by genetic factors and show relatively stable individual differences [45]. The differential susceptibility model states that some children are more sensitive to environmental factors and susceptible to adverse or favorable environmental factors due to temperament or genetics [46]. It has been shown that depression can act as a moderating variable in the influence of environmental factors on individual behavior or cognition [47,48].

IGD is usually the result of individuals using external means for self-regulation and experimenting with negative emotions [44]. Adolescents with higher levels of depression are more likely to seek external resources and experiences through online games because they have difficulty getting satisfaction in real life [49,50]. It has been demonstrated that depressive symptoms are a risk factor for IGD [44,49–51]. For example, Burleigh et al. showed that individuals with higher levels of depressed mood were more likely to exhibit IGD as a result of the type of game [44]. According to the comprehension theoretical model of problem behavior [24], IGD is the result of a dynamic interaction between risk and protective factors. Thus, depression may be a risk factor that increases the likelihood of adolescents developing problem behaviors when they are in specific developmental contexts. However, different researchers have held different views on the mechanisms by which depression affects individual behavior. Some researchers have suggested that "highly sensitive" individuals are both more likely to be overwhelmed by adverse circumstances and more likely to benefit from positive ones [45]. Other researchers have argued that for individuals with high negative emotions, simply improving their surroundings is not enough to modify their problem behavior because of their own high levels of negative emotions [48]. As middle school students are in an important period of physical and mental development, their psychological development is still at a semi-mature and semi-naive stage, and their psychological self-adjustment and recovery abilities are much lower than those of adults [52]. Therefore, we believe that individuals with high levels of depressed mood may still exhibit higher levels of IGD even if they possess high levels of PYD attributes. To our knowledge, the moderation effect of depression on the association between PYD and IGD needs to be deepened. Therefore, it is crucial to investigate how depression interacts with PYD in the formation of IGD in individuals.

### 1.4. The present study

**Fig 1:**
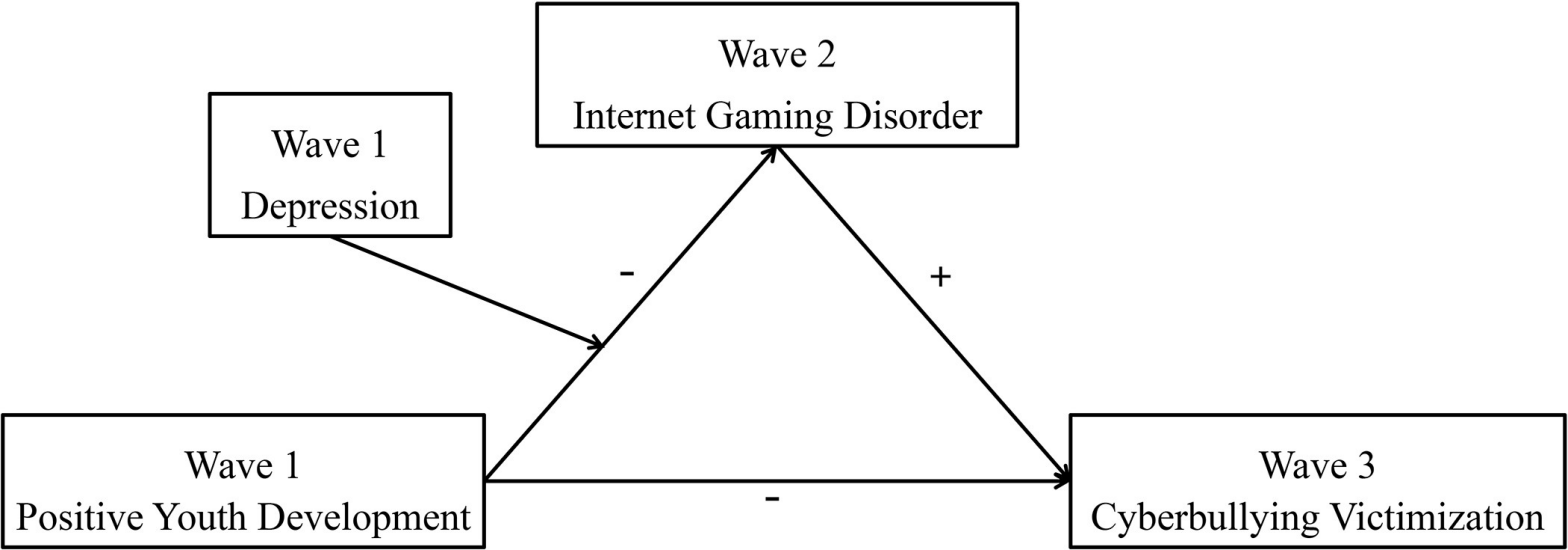
Proposed mechanism of the association between PYD and cyberbullying victimization.

To sum up, fewer studies have explored the potential influential mechanisms of cyberbullying victimization from a positive psychology perspective. Therefore, we aimed to examine the influence of PYD attributes, IGD, and depression on cyberbullying victimization and its internal mechanism in the same model, based on the above mentioned literature review and related theories. The hypothesis of the association mechanism between PYD attributes and cyberbullying victimization is as follows and the whole proposed model is depicted in Fig 1:

H1: PYD attributes can negatively predict cyberbullying victimization during middle school students;
H2: IGD will mediate the relationship between PYD attributes and cyberbullying victimization;
H3: Depression will moderate the pathway from PYD attributes to IGD.

Taken together, IGD and depression are expected to shed light on the relationship between PYD attributes and cyberbullying victimization. Identifying the influencing factors from cyberbullying victimization and the intrinsic dynamic influencing mechanisms have crucial implications for theory and prevention, providing new ideas for improving the physical and mental health of adolescents.

## 2. Methods

### 2.1. Participants and procedure

We recruited all grade 10 and grade 11 students as participants from a public school through clustering convenience sampling in Hubei Province, China. Following the voluntary principle, a total of 864 questionnaires were sent out, and 802 were successfully received, and the effective recovery was 92.8%. Due to voluntary withdrawal and transfer, 802 valid questionnaires were collected for Wave 1 (Nov. 2020), 746 valid questionnaires were collected for Wave 2 (May. 2021), but only 719 valid questionnaires remained for Wave 3 (Nov. 2021). The 719 (45.2% boy) valid middle school students tracked were between 14 and 18 years old (*M*_age_ = 15.95 years, *SD* = 0.76), and of these, 267 (37.1%) were grade 10 students and 452 (62.9%) were grade 11 students. Using G*Power 3.1.9.7 to calculate the sample size required for this study, it was found that 134–1289 samples were required to detect a small or medium effect size. Combined with related studies [53], our final sample size meets the requirements and can provide at least 95% statistical test power (1-*β*). Independent sample *t*-tests were used to compare the differences in the primary variables between the effective follow-up sample and the attrition sample at wave 1. The results showed that there were no significant differences between the two groups of participants in terms of PYD, IGD, and cyberbullying victimization levels (*p* > 0.05). However, the depression scores of the attrition sample were higher than those of the tracking sample (*t* = -2.10, *p* < 0.05).

The study was approved by the Research Ethics Committee of the Psychology, College of Education and Sports Sciences, Yangtze University (No. YZU20201014). A group test was conducted in the classroom, with graduate students trained in professional psychology acting as the conductor. Prior to conducting the test, the purpose of the study was explained to teachers and students, and the principle of data confidentiality was emphasized. All participants were told that they can quit at any time if they feel uncomfortable and were required to complete all items independently throughout the process. With the informed consent of students and their parents or legal guardians involved, the questionnaire survey was conducted. The questionnaire of the students who participated voluntarily was collected after they finished, while the remainder were not required to. We require students to complete the questionnaire within 20 minutes and thank them for their cooperation after completion.

### 2.2. Measures

#### 2.2.1 Positive youth development

At Wave 1, The Chinese Positive Youth Development Scale (CPYDS) developed by Shek et al. [54] was used, which contains 90 items. All items were rated on a 6-point Likert-type scale ranging from 1 (completely disagree) to 6 (completely agree). For example, "When I need help, I trust my parents to help me." The average score of all items is calculated (after reverse-coding when necessary), with higher scores indicating higher levels of PYD attributes. This scale has been widely used and has demonstrated good reliability and validity in China [55,56]. In the present study, Cronbach’s alphas coefficient was 0.974.

#### 2.2.2 Depression

At Wave 1, depression was measured with The Center for Epidemiological Studies Depression Scale (CES-D). This scale was compiled by Radloff [57] and used to measure depressive mood in the general population. In 1999, scholars translated the Chinese version of the CES-D [58], which is widely used to measure the level of depression in Chinese adolescents. The scale contains 20 items and all items were rated on a 4-point scale ranging from 0 (occasionally or not) to 3 (most of the time or continuously). For example, “I feel depressed”. Item scores were averaged to create a composite of depression, with higher scores indicating higher levels of depression. Studies have shown that this scale has good reliability and validity among Chinese adolescents [59,60]. In the current study, the scale’s Cronbach’s alphas coefficient was 0.765.

#### 2.2.3 Internet gaming disorder

At Wave 2, The questionnaire of Internet Gaming Disorder (IGD) compiled by Gentile [61] and revised by Yu et al. [31] was used, which contains 11 items. All items were rated on a 3-point Likert-type scale ranging from 1 (never) to 3 (often) to assess the frequency of each symptom in the last six months of the participant’s life. For example, “Do you need to spend more time on online games to get satisfaction?” The average score of all items is calculated, with higher scores indicating the higher tendency of IGD. This questionnaire has demonstrated good reliability and validity in samples of Chinese adolescents [62]. In the present study, the Cronbach’s alphas coefficient was 0.890.

#### 2.2.4 Cyberbullying victimization

At Wave 3, cyberbullying victimization was measured with 6 items complied by Lam and Li [15]. All items were rated on a 7-point scale ranging from 0 (never) to 6 (more than or equal to 6 times). The average score of all items was calculated, with higher scores indicating a higher frequency of suffering from cyberbullying. For example, “How many times did someone say mean things about you using emails, texting, short messages, on a website such as Renren, etc.?” Studies have shown that this questionnaire has good reliability and validity among Chinese adolescents [7,13]. The questionnaire’s Cronbach’s alphas coefficient was 0.924 in this study.

### 2.3. Statistical analysis

Prior research has found that adolescents’ gender and grade are related to cyberbullying [15,40,51,63]. Thus, we controlled for these demographic variables in our statistical analyses. The regression estimation method was used to fill in missing data. Because the data were collected through self-reporting, we conducted the Harman single factor method to test the common method biases. For descriptive statistics and correlation analysis, SPSS 26.0 was utilized. We used the SPSS macro PROCESS to examine mediation effect and moderating effect [64]. We used bootstrapping with 5000 iterations to test the statistical significance of the mediation effect. The interaction product was created by multiplying the predictor and the moderator. When an interaction effect was significant, simple slope analyses (1 *SD* above and below the mean of the moderator) were conducted [65].

## 3. Results

### 3.1. Common method bias analysis

Some approaches, such as reverse scoring and anonymous filling, were used to control the process to decrease the influence of the common method bias. Harman single factor test was used to inspect this bias [66]. The results reveal that the eigenvalues of 23 factors are more than 1, and the variance explained by the first factor is 25.73%, which is much less than the critical value of 40%, indicating that there is no significant common method bias in this study.

### 3.2. Descriptive statistics and correlation analyses

Means, standard deviations, and correlations are displayed in Table 1. As regard to covariates, boys had higher tendency of IGD (*r* = -0.28, *p* < .001) and frequency of suffering from cyberbullying (*r* = -0.09, *p* < .05) than girls. Students in higher grade had higher PYD level (*r* = 0.11 *p* < .01) and lower depression level (*r* = -0.10, *p* < .01) than lower grade. PYD are negatively correlated with cyberbullying victimization (*r* = -0.16, *p* < .001) and IGD (*r* = -0.24, *p* < .001), meaning that students who have higher levels of PYD attributes were less likely to suffer from cyberbullying behavior and develop IGD behavior. IGD is positively correlated with cyberbullying victimization (*r* = 0.16, *p* < .001), indicating that students with IGD are more likely to suffer from cyberbullying.

**Table 1:** Descriptive statistics and correlations for all variables.

Variables	*M*	*SD*	1	2	3	4	5	6
**1. Gender**	1.55	0.50	1					
**2. Grade**	4.63	0.48	0.05	1				
**3. PYD**	4.76	0.66	-0.04	0.11**	1			
**4. Depression**	1.86	0.37	0.03	-0.10**	-0.28***	1		
**5. IGD**	1.38	0.37	-0.28***	-0.07	-0.24***	0.15***	1	
**6. CV**	0.28	0.72	-0.09*	-0.02	-0.16***	0.11**	0.16***	1

Note: Use 1 for boy and 2 for girl; Use 4 for grade 10 and 5 for grade 11. PYD Positive Youth Development, IGD Internet Gaming Disorder, CV Cyberbullying Victimization. ***p < .001, **p < .01, *p < .05. the same below.

### 3.3. Mediating effects of internet gaming disorder

**Fig 2:**
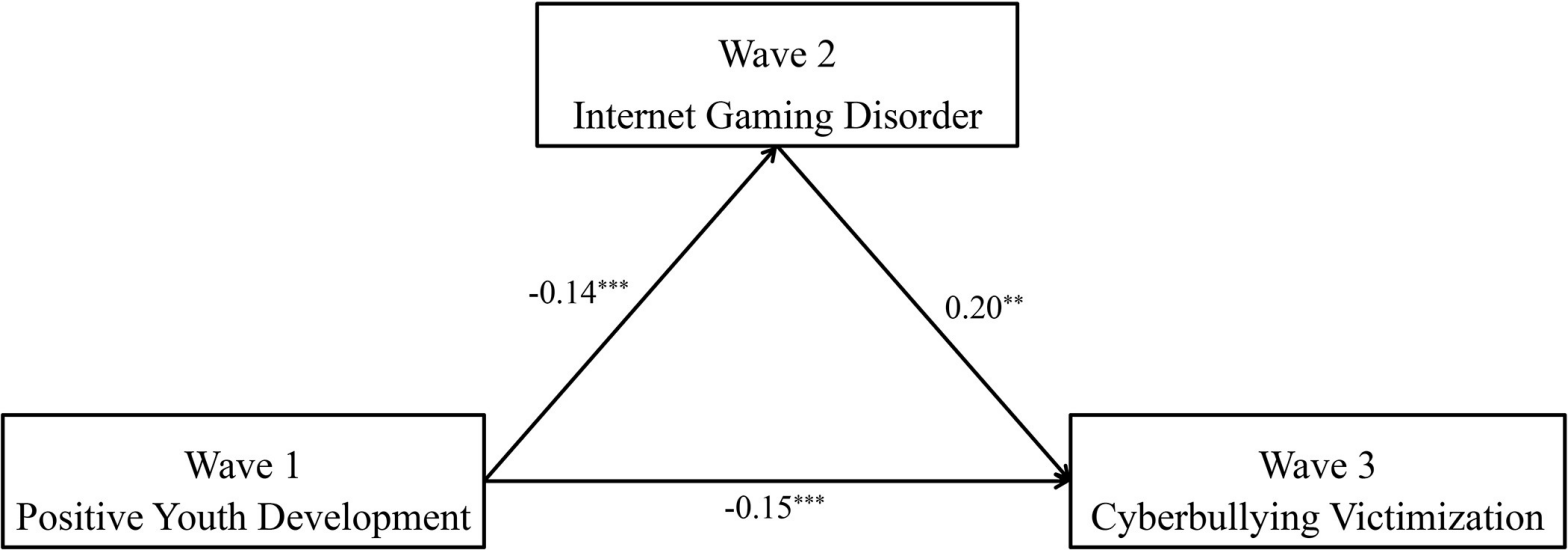
The mediating role of IGD. All covariates were held constant during this analysis but are not presented for reasons of simplicity.

Model 4 of the SPSS macro program PROCESS was used to test the mediating effect of IGD. The results are shown in Table 2. After controlling for gender and grade, PYD attributes is a significant negative predictor of cyberbullying victimization (*β* = -0.18, *p* < .001), and H1 1 is proved. When both PYD and IGD enter the regression equation, PYD can significantly negatively predict cyberbullying victimization (*β* = -0.15, *p* < .001), and significantly negatively predict IGD (*β* = -0.14, *p* < .001). IGD can significantly positively predict cyberbullying victimization (*β* = 0.20, *p* < .01). The bias correction Bootstrap method test shows that IGD has a significant mediating effect between PYD attributes and cyberbullying victimization, *ab* = -0.03, 95% *CI* [-0.063, -0.001], proving H2. As shown in Fig 2, the mediation effect accounted for 16.67% of the total effect of PYD attributes on cyberbullying victimization.

**Table 2:** The mediating model of PYD and cyberbullying victimization.

Variables	Model 1 CV		Model 2 IGD		Model 3 CV	
	*β*	*T*	*Β*	*t*	*β*	*t*
**Gender**	-0.14	-2.70**	-0.22	-8.37***	-0.10	-1.80
**Grade**	-0.001	-0.03	-0.02	-0.87	0.003	0.06
**PYD**	-0.18	-4.36***	-0.14	-7.02***	-0.15	-3.57***
**IGD**					0.20	2.63**
***R*** ^***2***^	0.04		0.14		0.04	
***F***	8.60***		39.67***		8.23***	

### 3.4. Moderating effects of depression

**Fig 3:**
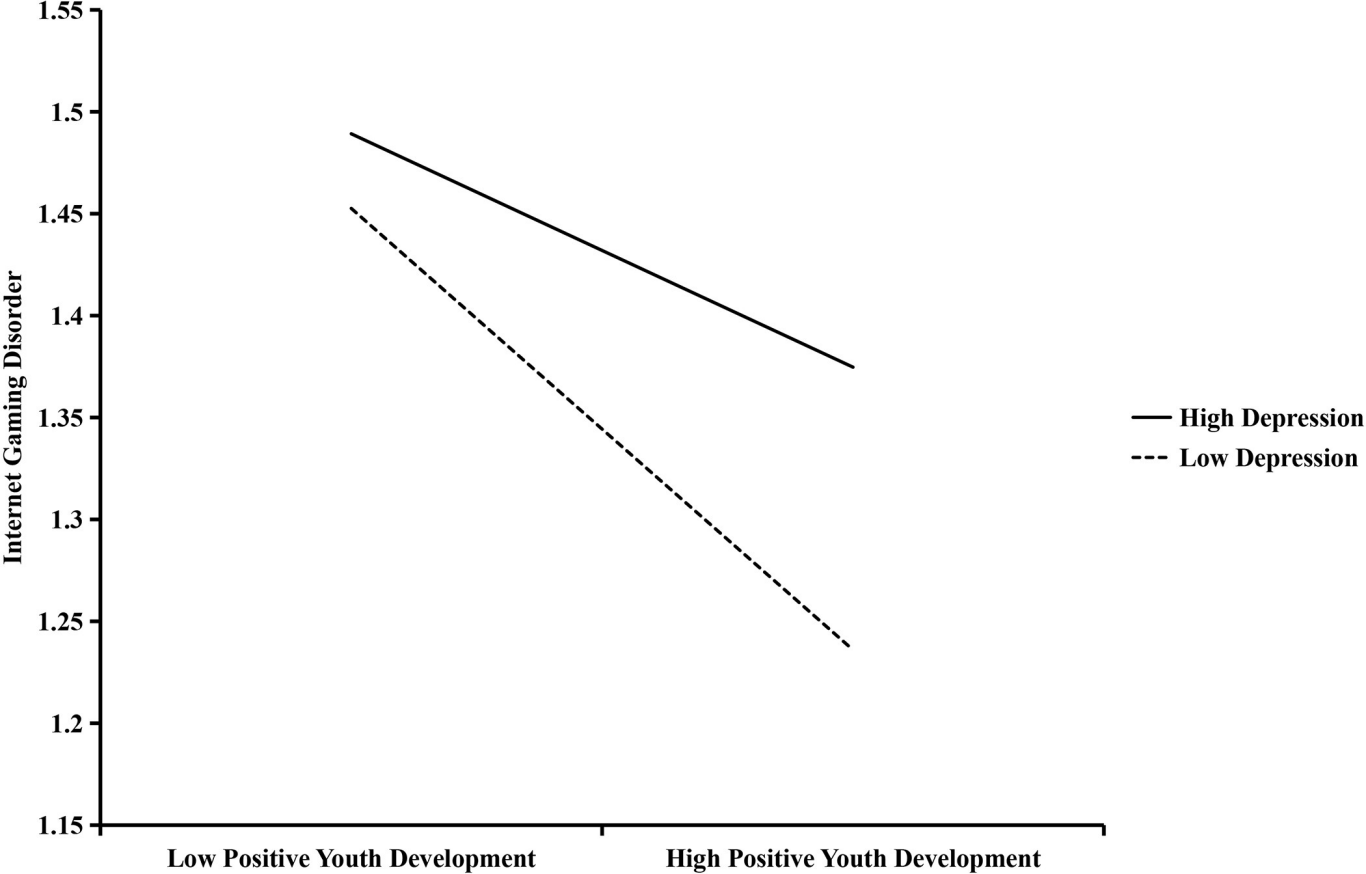
The moderating role of depression in the effect of PYD on IGD.

Model 7 of the SPSS macro program PROCESS was used to test the moderating effect of depression. The results are shown in Table 3, PYD significantly negatively predicted IGD (*β* = -0.12, *p* < .001), and the interaction term between PYD and depression significantly positively predicted IGD (*β* = 0.10, *p* < .05), meaning that depression moderated the relationship between PYD attributes and IGD, proving H3. Follow-up analyses (presented in Fig 3) tested simple slopes of PYD at lower (1 *SD* below mean) and higher (1 *SD* above mean) levels of depression to understand the nature of the interaction. For students with a high level (-1 *SD*) of depression, PYD attributes was associated with higher IGD. For students with a low level (+1 *SD*) of depression, the relationship between PYD attributes and IGD was relatively stronger. Thus, higher depression seemed as a risk factor while lower depression was a protective factor in the association between PYD attributes and IGD.

**Table 3:** The moderating model of PYD on IGD.

Variables	*Β*	*T*	*SE*	*LLCI*	*ULCI*
**Gender**	-0.22	-8.46***	0.03	-0.27	-0.17
**Grade**	-0.02	-0.73	0.03	-0.07	0.03
**PYD**	-0.12	-6.20***	0.02	-0.16	-0.09
**Depression**	0.12	3.17**	0.04	0.05	0.19
PYD * Depression	0.10	2.08*	0.05	0.006	0.20
***R*** ^***2***^	0.16				
***F***	26.47***				

## 4. Discussion

This study used a longitudinal research design to explore the factors influencing cyberbullying victimization and its potential internal mechanisms from a positive psychology perspective. The findings suggest that PYD attributes is an important factor in explaining cyberbullying victimization among middle school students, and that it influences cyberbullying victimization directly or indirectly through IGD. Moreover, the relationship between PYD attributes and IGD was also moderated by the level of individual depression.

The results of the descriptive statistical analysis revealed that the IGD levels and cyberbullying victimization levels were significantly higher in boys than in girls, which has similarities with the results of previous studies [67,68]. This may be because boys’ brains are more sensitive to stimuli that combine light, sound and moving images, and therefore more likely to get unique experiences from online games [67]. For cyberbullying victimization, on the one hand, boys are more likely to be victims of cyberbullying because they have higher levels of IGD and have more access to the internet. On the other hand, it may be because girls are socially recognized as a protected group and thus are also less likely to be victims of bullying [69]. In addition, the results of the study showed that students in higher grades had higher levels of PYD attributes and lower levels of depression, which is similar to previous studies, but also inconsistent [70,71]. Such results may be related to the small range of subjects we selected, and the fact that the PYD attributes of the students may have changed due to the influence of the entrance examination.

The findings demonstrate that the higher the level of PYD, the lower the adolescents’ cyberbullying victimization level, which is in accordance with related findings [16,17,25] and also makes extension to it. Conformity positive youth development theory [18] and the comprehension theoretical model of problem behavior [24], this study consider cyberbullying victimization in a more comprehensive system. In this sense, individuals with higher levels of PYD attributes achieve positive individual development and reduce problem behaviors due to the availability of sufficient external and internal resources. The development assets theory similarly states that problem behaviors may occur due to a lack of developmental assets, such as positive psychological resources [72]. Longitudinal findings better illustrate that cyberbullying victimization can be reduced in subsequent online environments by enhancing students’ levels of PYD attributes. These finding improved our understanding of cyberbullying victimization and lay the foundation for more research into the mediating roles of IGD and moderating roles of depression in the connection between PYD attributes and cyberbullying victimization.

Our findings suggest that PYD is a negative predictor of IGD, which in turn increases the likelihood that students will be victims of cyberbullying. This finding also suggests that IGD may be an important explanatory mechanism for the link between PYD attributes and cyberbullying victimization. The mediating effect of IGD is consistent with the results of previous studies [16,25]. In addition, this study builds on previous research and deepens our understanding of the mechanisms influencing of IGD. According to developmental contextualism [42], PYD not only directly influences cyberbullying victimization but may also influence cyberbullying victimization in conjunction with the internet context. Of these, the level of PYD attributes reflects both the external resources for individual growth and can demonstrate internal motivation for adolescent development. For individuals with low levels of PYD attributes, internet games provide a range of external resources [62]. At the same time, adolescents with IGD spend a disproportionate amount of time in the internet environment. Thus individuals’ PYD traits increase the likelihood of cyberbullying victimization through the internet environment they are in. However, it is worth noting that IGD plays a partial mediating role in the relationship between PYD and cyberbullying victimization, indicating that the influence of PYD on cyberbullying victimization may be mediated by other factors, and extensive research is needed.

As expected, depression moderated the pathway from PYD to IGD. To be specific, individuals with higher levels depression were more likely to develop IGD than those with lower levels of depression. That is, depression is a risk factor for IGD. However, this result is not entirely consistent with previous studies [73–75]. The researchers concluded that it was internet gaming or even problematic internet gaming behavior that significantly predicted an individual’s psychological quality of life [76], distress (e.g., depression) [77]. However, there are other researchers who support the reverse path. They suggest that psychological distress (e.g., depression) is an antecedent of problematic use of internet-related activities [78] and can significantly predict problematic gaming [79]. Chang et al. demonstrated that internet gaming may be a coping strategy for individuals with schizophrenia through their study of participants diagnosed with schizophrenia [80]. Kakul and Javed, based on clinical model, also showed that individuals with high IGD levels may subscribe to a unique set of maladaptive beliefs that underlie persistent and excessive engagement in internet gaming activities. Therefore, we believe that students with higher levels of depression are more inclined to experience negative emotions and fulfill desires that are difficult to satisfy in real life through the internet environment [59,60]. So reducing students’ depression levels is of great importance to fully utilize PYD resources. At the same time, we should also note that although individuals with higher levels of depression have higher levels of IGD at the same level of PYD, their PYD attributes are relatively more weakly associated with IGD. That is, the favorable impact of the PYD attributes shrinks significantly when individual risk is high. According to this result, improving PYD attributes most benefited individuals with lower levels of depression, while lowering depression levels most benefited individuals with higher levels of PYD. In addition, future research needs to explore additional risk factors and protective factors to better leverage the strengths of adolescents and reduce cyberbullying victimization.

## 5. Contributions and limitations

There are some important contributions of this study. First, this study examines the impact of PYD attributes on cyberbullying victimization from a positive psychology perspective, and the underlying mechanisms. This finding clarifies the importance of PYD attributes in students’ deviant behaviors, rather than continuing to understand their behavioral changes in terms of managing problem behaviors. At the same time, exploring adolescent cyberbullying victimization from the perspective of intrinsic mechanisms rather than impact effects not only provides empirical support for positive youth development theory, but also offers new perspectives on the prevention and intervention of adolescent problem behaviors. Therefore, in the subsequent educational process, we should focus on creating an environment conducive to the positive development of students and enhancing their own abilities so that they can be free from cyberbullying victimization. Second, the mediating role of IGD suggests that intervention programs can focus on reducing students’ time spent online and further improving mental health. In addition, the moderating effect of depression suggests that by reducing depression levels in adolescents, students can reduce the occurrence of IGD in specific settings, thereby reducing cyberbullying victimization. Although PYD attributes are protective factors for IGD, the effect of this protective factor is significantly diminished when individuals have high levels of depression. Therefore, for reducing individual depression levels can significantly improve the IGD levels of individuals with high PYD.

Despite the above-mentioned contributions, some limitations of this study should be mentioned. First, this study examined the dynamic relationships between variables using a longitudinal research design, but little attention was paid to the interactions between variables. It is expected that future studies will examine the interactions between variables and use more advanced statistical methods (e.g., cross-lagged panel models) for analysis. Second, we used the self-reporting method to collect data, which may be affected by social desirability. Future research should use multiple methods and multiple informants to increase the validity of the findings. Third, this study found that the relationship between PYD attributes and cyberbullying victimization was partially mediated, suggesting that a further understanding of this relationship requires information on other mediators. Fourth, research has identified the complex nature of the bullying experience, in that some bullies are also victims. Whether cyberbullying shares this characteristic and the mechanisms underlying such group behavior is also something we need to explore in the future. Finally, the current study was conducted with a sample of Chinese middle school students, and again, the measurement instrument has only been shown to have good reliability in the Chinese cultural context (e.g., the questionnaire of Internet Gaming Disorder revised by Yu et al. [31]). Future studies should include cross-cultural samples of adolescents, using international scales with good psychometric properties (e.g., the nine-item Internet Gaming Disorder Scale-Short Form [81–84]), in order to test the generalizability of our findings.

## 6. Conclusion

This study explores the mediating role of IGD and moderating role of depression in PYD attributes and cyberbullying victimization, and draws the following conclusions: PYD attributes could negatively predict cyberbullying victimization; The relationship between PYD attributes and cyberbullying victimization is mediated by IGD; The relationship between PYD attributes and cyberbullying victimization is moderated by depression.

## Supporting information

S1 Dataset Dataset used for analyses in present study. (SAV)
